# A role for pre-mNK cells in tumor progression

**DOI:** 10.1186/s40425-016-0120-6

**Published:** 2016-03-15

**Authors:** Carolyn Rosinsky, Paul Andrew Antony

**Affiliations:** Program in Molecular Medicine, University of Maryland School of Medicine, Baltimore, MD 21201 USA; Department of Pathology, University of Maryland School of Medicine, Baltimore, MD 21201 USA; Department of Microbiology and Immunology, University of Maryland School of Medicine, 10 South Pine Street, 734D MSTF, Baltimore, MD 21201 USA; Tumor Immunology and Immunotherapy Program, University of Maryland Cancer Center, Baltimore, MD 21201 USA

**Keywords:** Pre-mNK cells, IKDC, CD4^+^ T cells, Melanoma, PD-L1

## Abstract

The innate and adaptive immune systems have evolved together to fight infection and cancerous tissues. The innate immune system emerges first with the adaptive immune system following, both ostensibly being bridged by dendritic cells (DC). Recently cells have emerged that possess characteristics of both innate and adaptive immune cell qualities, termed interferon-producing killer dendritic cells (IKDCs). These cells have an indistinct origin that is not well understood. They appear to have more NK cell attributes than DC but purportedly can regulate the immune system similar to immunoregulatory NK cells. Because of this, they have been renamed pre-mNK cells (pre-mature NK cells). We argue in this commentary that pre-mNK cells may contribute to cancer recurrence.

## Introduction

**Fig. 1:**
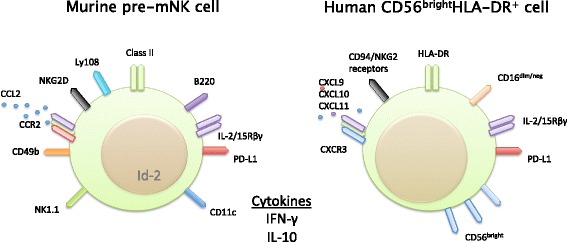
Comparison between the immune-related molecules expressed on murine pre-mNK cells and human CD56^bright^ HLA-DR^+^ NK cells. Murine pre-mNK cells classically express CD11c^lo^CD49bB220NK1.1 and are GR-1^neg^ in C57BL6/mice, but lack NK1.1 in other strains. Pre-mNK cells also express NKG2D and respond to the chemokine CCL2 due to expression of CCR2, making them apt for migrating to tumor sites. Upon licensing by tumor cells, pre-mNK cells express class II and other immune related molecules. The Id-2 transcription factor is prevalent in pre-mNK cells showing that they are more NK cell-like than DC. Both murine and human CD56^bright^HLA-DR^+^ NK cells express the IL-2/IL-15Rβγ, making them responsive to these cytokines in different contexts. Human CD56^bright^HLA-DR^+^ NK cells express CD56 at high levels, HLA-DR, and CD94/NKG2 receptors, and low to no amounts CD16. They are CXCR3 expressing cells making them able to migrate to secondary lymphatic tissues or places of inflammation. Both murine and humans cells may also express PD-L1 in different environmental conditions, and both can make IFN-γ and IL-10 in varying amounts, again depending on their environment. By no means are these molecules listed complete or absolute and further research is needed to clarify the roles of each on these cells

Pre-mature Natural Killer cells (pre-mNK) are murine hybrid cells with characteristics of both NK cells and DC, defined by markers CD11c^+^CD49b^+^B220^+^NK1.1^+^NKG2D^+^GR-1^−^ and expressing MHC class II upon activation [1–4] (Fig. 1). Originally called IKDC, these cells were first characterized in 2006 [1, 5, 6], but since then have been recognized to more closely resemble NK cells rather than DC or plasmacytoid DCs (pDCs) [7, 8]. Specifically, pre-mNK cells resemble an immature NK, before the cell begins to express CD27, and have classical attributes of cellular immaturity such as immature cell morphology, expression of Ly108, and low amounts of CD43. These cells also are dependent on the Id-2 transcription factor, which has been shown to inhibit pDC differentiation [9]. Once these cells begin to express CD27, they stop expressing the pre-mNK marker B220 and their function changes, commonly from that of a pre-mNK cell to that of a mature NK (mNK) cell [6–8, 10]. Little is known about the formation or differentiation of these cells, but their development is IL-15 dependent [2, 4, 11], and appears to depend on their environment in which they reside and become activated [6, 12]. Below we discuss their implications in tolerance to tumors both in mice and humans.

### Murine pre-mNK cells

Initially, murine pre-mNK cells were described in mice to have anti-tumor properties [5, 6]. However, subsequent studies, including our own data, have shown that depletion of NK1.1^+^ cells leads to enhanced tumor rejection, viral clearance, and decreased exhaustion of T cells [13–19]. Surprisingly most of the studies that depleted NK1.1^+^ cells failed to show that pre-mNK cells were also being depleted [11]. These findings seem to contradict the anti-tumor, cytotoxic role of these cells. One recent study showed that activated pre-mNK cells prevented autoimmunity through PD-L1 and IL-10 [20]. Therefore, it seems that the natural predilection for pre-mNK cells is to suppress autoimmunity. We hypothesize that this attribute of pre-mNK cells contributes to tumor recurrence.

The role of pre-mNK cells in the context of immunotherapy using adoptive cell transfer (ACT) of T cells has not been studied. Pre-mNK cells have mainly been used in isolation as tumor killers or in the context of non-self tumor-antigens such as OVA. These non-self antigens activate high affinity T cells that were not educated for OVA in the thymus, and as a result, T_reg_ cells to OVA do not exist [21]. This may confound the natural activity of pre-mNK cells which is to suppress tumor-antigens that are also self-antigens.

Previously, we have shown that CD4^+^ T cells specific for a tumor-associated self-antigen (TAA) called tyrosinase-related protein 1 (TRP-1), a melanoma differentiation antigen (MDA), can treat large established tumors by direct killing of cancerous cells [16]. We found that lymphopenia (either induced before adoptive transfer or naturally occurring in RAG^−/−^ mice) enhanced rejection of tumors through loss of regulatory elements such as T_reg_ cells and MDSCs [22] or through increased homeostatic cytokines that could potentially help T cells attack tumors better [23, 24]. Nevertheless, about 50 % of the tumors would recur locally [23, 24]. Surprisingly, cancer recurrence was diminished considerably when NK1.1^+^ cells were depleted with PK-136 depleting antibodies. In addition, depletion of NK1.1^+^ cells increased autoimmune vitiligo, serum inflammatory cytokines, and chemokines [11]. This was thought to be due to the absence of NK cell “cytokine sinks,” [22] thus enhancing the cytotoxicity of the CD4^+^ T cells through increased availability of IL-2 and possibly IL-15. However, depletion of NK cells with NK cell-specific antibody, called asialo-GM1, failed to fully duplicate the results from experiments employing NK1.1^+^ cell depletion [11]. Furthermore, we showed that B220^+^ cell depletion but not asialo-GM1^+^ cell depletion was similar to the depletion of NK1.1^+^ cells, suggesting that B220^+^NK1.1^+^ pre-mNK cells were playing a role in dampening the CD4^+^ anti-tumor response in our preclinical model of melanoma [11]. These data made us question the purported role of pre-mNK cells in cancer biology.

Since 2006, the role of pre-mNK in the tumor setting has been equivocal. Taieb et al. first described that treating melanoma with Imatinib and IL-2 resulted in pre-mNKs expanding in the spleen, producing copious IFN-γ, and killing tumor cells via TRAIL [5]. Although the tumor microenvironment affects the function of pre-mNK [8], only our group studied pre-mNK cells without ex vivo pre-activation, calling into question the anti-tumor role of pre-mNK cells in the *natural course* of the disease [11]. We suggest, as others have implicated, that pre-mNK cells must be licensed by tumor cells through NKG2D or other ligands to become activated [3, 21]. This leads to their maturation into functional APCs through upregulation of MHC class II and other receptors, and their migration into draining lymph nodes where they present tumor-self antigens to self-reactive T cells which are tolerized or programmed to become T_reg_ cells rather than activated effector cells. Experiments by others using blocking antibodies to NKG2D decreased the activation of OTII cells by OVA expressing B16-Rae1 cells [21], suggesting that if licensing cannot occur by tumor cells, pre-mNK cells cannot interact with T cells through MHC class II. Licensing occurs to allow pre-mNK cells to kill tumor cells initially so that they may acquire tumor antigen for presentation, occurring in less than 48–72 h as shown by migration experiments [3]. This is temporary and results only in delay of tumor growth [3]. We suggest that this *licensing* could be mistaken for tumor killing.

Although pre-mNK cells were first described for their role in anti-tumor immunity, they have been described as controlling tolerance to self-antigens [20]. These cells prevent autoimmunity or reduce the severity of autoimmune conditions such as experimental autoimmune encephalitis (EAE) [4, 17]. Melanoma has also been shown to license NK1.1^+^B220^+^CD11c^+^MHC class II^+^ pre-mNK cells to present tumor antigens [3, 21]. Pre-mNK cells also express the inhibitory PD-1 ligand (PD-L1) [12, 20, 21] and can make the immunosuppressive cytokine, IL-10 [20]. PD-L1 has clearly been shown in pre-clinical [25–28] and clinical scenarios to inhibit tumor immunity through adaptive resistance mechanisms [29, 30] as well as to be involved in controlling chronic infections and autoimmunity [31–35]. Thus pre-mNK cells expressing PD-L1 could potentially suppress immunity to cancer, like cancer cells themselves. Because melanoma can expresses TRP-1, a melanocyte differentiation antigen expressed in the skin that is targeted by our TRP-1 specific CD4^+^ T cells, autoimmunity can ensue. Therefore, pre-mNK cells might be involved in tolerance rather than immunity during an immune response to melanoma. This is demonstrated in our recent work showing that autoimmune vitiligo is increased in tumor-bearing mice treated with CD4^+^ T cells specific for TRP-1 and antibodies to deplete NK1.1^+^ cells [11].

Like NK cells, pre-mNK cells are IL-15 dependent [1]. It has been shown that production of hIL-15 by in vivo gene transfer in mice increases pre-mNK cell numbers and function [36]. On the contrary, IL-15^−/−^RAG^−/−^ and IL-2Rγ^−/−^RAG^−/−^ mice both lack NK and pre-mNK cells [4, 7, 11]. Consistent with this, we have shown that tumor rejection is enhanced in IL-15^−/−^RAG^−/−^ mice and that recurrence of melanoma is less when compared to IL-15 wild type controls [11]. In line with this data, it has been shown that pre-mNK cells are low in autoimmune prone NOD mice, which are susceptible to diabetes due to genetic mutations linked to the distal end of chromosome 7 [37]. If the distal end of chromosome 7 is modified to express WT genes as in NOD-Lc7 mice, they are not prone to diabetes, and have restored numbers of pre-mNK cells [1]. This suggests that the natural tendency of pre-mNK cells is to cause suppression rather than immunity in vivo.

Pre-mNK cell function modulates over the course of an immune response. After stimulation, presumably through NKG2D and other ligands, pre-mNK cells first acquire lytic activity, signaling through classical NK receptors such as NKG2D, and killing through NKG2D and TRAIL [3, 6, 10]. They then produce IFN-γ, and increase expression of MHC-II and migrate via chemokine receptors to the secondary lymphatic tissue [6, 10]. In the tumor microenvironment, direct contact with the tumor cell provides this stimulation, leading not only to MHC-II expression and IFN-γ production, but also to IFN-γ-induced PD-L1 expression [8, 12, 21, 30]. We suggest that this adaptive immune response induces exhaustion and increases T_reg_ cells, suppressing T cell function. Because the tumor cell itself licenses pre-mNK cells for antigen presentation, the antigens encountered are likely to be self-antigens [3, 21]. Thus, in untreated cancer, *unmanipulated* pre-mNK cells may act to protect the host by dampening the immune response — beneficial in autoimmunity, but detrimental in cancer or chronic infections [20]. However, *experimentally manipulated* pre-mNK cells, those which are mostly studied up to this point, may actually activate the immune response because treatment-induced cellular trauma makes more DAMPs available, thus confounding their natural role [1, 3–7, 21].

### Human pre-mNK cells

The closest pre-mNK analog in humans is the CD56^bright^ NK cell [38] or the HLA-DR^+^ NK cell, which is a subset of CD56^bright^ NK cells [39]. Here, we will refer to the human equivalent as CD56^bright^HLA-DR^+^ NK cells because these cells expand only from HLA-DR^+^ NK cell populations and can present antigen [39]. However, we will also refer to CD56^bright^ cells alone when discussing literature pertaining only to them and try to draw out similarities between CD56^bright^, CD56^bright^HLA-DR^+^ NK cells, and mouse pre-mNK cells. Our goal is to suggest that pre-mNK cells in humans are CD56^bright^ HLA-DR^+^ NK cells.

Like pre-mNK cells, CD56^bright^ NK cells are recognized as an immunoregulatory NK subset in humans [40–42], and are defined by the markers CD3^−^CD56^+^CD16^dim^HLA-DR^+^ [38, 39, 42–45] (Fig. 1). Like pre-mNK cells they represent a small subset of total NK cells, approximately less than 10 % of CD56^+^ cells [42]. In their regulatory role, they lyse CD4^+^ T cells via TRAIL and NKG2D, and secrete large amounts of IFN-γ and regulatory cytokines [41, 45, 46]. Like pre-mNK cells, CD56^bright^ NK cells are dependent on IL-15 for development and activation [42–44], and are implicated in controlling autoimmunity and mediating the immune response to cancer and viral infections. In non-pathologic physiology, CD56^bright^ NK cells maintain fetal tolerance by inhibiting Th-17-mediated immune responses at the maternal-fetal interface [47].

HLA-DR^+^ NK cells also expand to IL-15 and high doses of IL-2 [39]. However, these types of experimental systems used to study these cells in vitro, we would argue, can be highly non-physiologic and may drive HLA-DR^+^ NK cells to a cytotoxic phenotype, leading to large amounts of IFN-γ secretion. However, their physiological role in vivo without external activation may involve tolerance mechanisms as these cells are seen at sites of inflammation, cancer, and at the maternal-fetal interface [47–52].

Solid tumors have large populations of CD56^bright^ NK cells at the primary tumor bed and at metastases, and similarly to pre-mNK, they expand following treatment [48–52]. As tumors progress or metastasize, CD56^bright^ NK cells remain present in the primary bed, at the metastases, and in lymph tissue, but lose function or become inactive despite high expression levels of perforin and HLA-DR [50, 53, 54]. These cells we hypothesize are CD56^+^HLA-DR^+^NK cells mentioned above that have induced tolerance to tumor.

CD56^bright^ NK cells have lytic activity via TRAIL, and when activated, upregulate HLA-DR and IFN-γ production to present antigen and modulate the immune response, either productively or pathologically [55]. This may be similar to pre-mNK cell activation through NKG2D ligands on murine tumors, allowing them to present antigen once it is acquired [3]. Although they can be lytic, it also has been shown that in both the viral and the autoimmune setting that CD56^bright^ NK cells control CD4^+^ T cell activity by expressing high levels of CD39/73 and CD38, using adenosine as a modulator of T cell activity. Stelma et al. report that after HBV treatment and improved ALT, the host’s CD56^bright^ NK cells express high levels of CD38, attenuating the chronic pathologic immune response [55]. Morandi et al. report that CD38 inhibition increases Juvenile Idiopathic Arthritis (JIA) severity, while the CD38 enzyme expressed by CD56^bright^ NK cells in patients with active JIA has attenuated function [56].

## Conclusion

CD56^bright^ HLA-DR^+^ immunoregulatory NK cells are potentially the human analog of the contentious murine pre-mNK cell. These highly proliferative cells, though a small population in both species, can readily expand to regulate the immune response, acting through direct cytolysis, cytokines, and metabolic signals, and acting as antigen-presenting cells with the capability to activate or terminate an immune response. These cells in human and mouse clearly stifle immune activity in autoimmunity and pregnancy. Their role in chronic pathology is more complex. Acting through cytolysis and/or antigen-presentation, CD56^bright^ HLA-DR^+^ NK or pre-mNK cells may dampen the response to chronic low-level stimulation or self-antigen, for instance during established melanoma, but they activate a response under acute conditions of treatment-induced DAMPs, high-level PAMP stimulation, or high doses of γ_c_-cytokines such as IL-2 or IL-15. Thus, pre-mNK or CD56^bright^ HLA-DR^+^ NK cells have the potential to be exploited therapeutically, but their opposing roles in differing immune environmental milieus must be taken into consideration.

CD56^bright^ cells and murine counterpart pre-mNK cells represent potential targets for immunotherapies, whether to suppress the immune system to prevent autoimmune diseases, or to enhance the immune system to treat cancer. It is clear that more research is needed to fully elucidate the role of these cells during an immune response in human disease.

## Abbreviations

ACT: Adoptive cell transfer
DAMPs: Damage associated molecular patterns
DC: Dendritic cell
IDO: Indoleamine-2,3-deoxygenase
IL: Interleukin
IKDC: Interferon killer dendritic cell
INF-γ: Interferon γ
NK cell: Natural killer cell
PAMPs: Pathogen associated molecular patterns
PD-1: Programmed cell death protein 1
PD-L1: Programmed death ligand 1
pDC: Plasmacytoid Dendritic Cell
Pre-mNK cells: Pre-mature natural killer cells
TLR: Toll-like receptors
T_reg_: Regulatory T cells
TRP-1: Tyrosinase related protein 1
